# Morphometric Study of the Inferior Alveolar Canal in a Sudanese Population

**DOI:** 10.7759/cureus.80194

**Published:** 2025-03-07

**Authors:** Rabah Mahjoub A AbdElrahman, Abbas Gareeb A Abdalla, Yasir H Elhassan, Syed Nazar Imam, Ghada A Wassif, Shabina Anjum Mohammad Jabir

**Affiliations:** 1 Department of Anatomy, National University, Khartoum, SDN; 2 Department of Anatomy, University of Khartoum, Khartoum, SDN; 3 Department of Basic Medical Sciences, College of Medicine, Taibah University, Madinah, SAU; 4 Department of Anatomy, College of Medicine, Taibah University, Madinah, SAU

**Keywords:** cbct, inferior alveolar canal, mandible, morphometry, sudanese population

## Abstract

Background and aim

The inferior alveolar canal (IAC) may be positioned at different locations in the mandible. Understanding its morphology is essential to avoid iatrogenic injuries to the neurovascular bundle during mandibular surgeries and dental procedures. Cone-beam computed tomography (CBCT) is a crucial imaging technique that provides visualization and presurgical measurement of the IAC and other maxillofacial structures. The aim of this study was to determine the morphometric analysis of the IAC in a Sudanese population using CBCT.

Materials and methods

This descriptive-analytical cross-sectional study was conducted in the implantology center from 01.03.2022 to 31.03.2023. Ethical approval was obtained, and informed consent was taken. A total of 100 subjects were selected, and they all underwent CBCT before planning treatment. All Sudanese patients in the age range of 18 years to 65 years were included, while all non-Sudanese patients and patients with preexisting mandibular diseases or trauma were excluded. The Blue Sky Plan software (Blue Sky Bio LLC, Grayslake, US) was used to measure all the parameters on CBCT. The vertical dimension of the mandible surrounding the IAC (above and below the canal) on both the right and left sides, as well as the horizontal dimension of the mandible surrounding the IAC (buccal and lingual) on both the right and left sides of the mandible, was noted. For comparison between the genders, the following parameters were measured vertically: in the ramus - ramus vertico apical (VA), ramus vertico occlusal (VO); in the body - body VO, body VA; and in the mental area - mental VO, mental VA. The following horizontal dimensions were also measured: in the ramus - ramus horizontal lingual (HL), ramus horizontal buccal (HB); in the body - body HB, body HL; and in the mental area - mental horizontal. The data were analyzed using the IBM SPSS Statistics version 20.0 (IBM Corp., Armonk, US).

Results

Of the 100 Sudanese patients, 84% were female and 16% were male. According to gender, we discovered a significant difference (p=0.003) in ramus HB on the right side, but a statistically insignificant difference (p=0.443) on the left side. The ramus VO on the left side also revealed a significant gender difference in male and female patients (p=0.039). Gender-specific comparisons of the ramus VO on the right side revealed no differences (p=0.175). Similarly, the difference between the right and left side ramus VA according to gender was not statistically significant (p=0.829, p=0.07). Other parameters such as body VO, body VA, and ramus HL did not differ significantly in statistical terms between the two genders.

Conclusion

This study investigated the morphometric analysis in various mandibular measurements in a Sudanese population. Most of the morphometric parameters were similar on both sides of the mandible and showed no significant differences in the male and female populations. Ramus HB on the right side and ramus VO on the left side revealed significant gender differences, indicating that these parameters could be helpful in gender determination in case of missed identity, especially in forensic cases for the Sudanese population.

## Introduction

The inferior alveolar canal (IAC) is the pathway for the inferior alveolar neurovascular bundle in the mandible. The well-known term for this bony canal is the mandibular canal; additionally, the term inferior alveolar nerve canal is accepted in many scientific research journals [1,2]. The human IAC is a passage within the mandible that begins in the mandibular foramen on the medial surface of the ascending mandibular ramus, runs obliquely downward and forward in the mandibular ramus, then horizontally forward in the body of the mandible, and ends where the mental foramen opens. It provides entry to the inferior alveolar nerve and vessel through the mandibular foramen and exits to the mental nerve and vessels through the mental foramen [3]. The IAC splits into the mental and incisive canals [4]. The incisive canal continues forward to the incisive teeth, whereas the mental canal runs upward and backward to the mental foramen, which opens below the second premolar or the space between the first and second premolars. These canals carry the incisive and mental branches of the inferior alveolar nerve and vessels, respectively [5].

The IAC, containing the inferior alveolar nerve and vessels, is crucial for dental and surgical procedures. Understanding its morphology is essential to avoid complications like nerve damage during surgical treatment in the mandible, such as mandibular implants, as well as other surgical procedures like mandible fracture. It is important to determine the location of the IAC before surgery to prevent vascular trauma or damage to the inferior alveolar nerve. Injury to this crucial nerve may lead to paresthesia of the lower lip and mentalis muscle area. Also, bone healing around the dental implant may be impaired if the implant comes in contact with the soft tissues lining the inferior alveolar nerve and vessels, which might lead to instability of the implant [6-8].

Computed tomography (CT) dental reformatting programs have become state-of-the-art for evaluating lesions of the jaw and for assessing dental implant patients [9,10]. The introduction of cone-beam computed tomography (CBCT) for the maxillofacial region provides opportunities for dental practitioners to request multiplanar imaging. CBCT allows the creation in “real-time” of images not only in the axial plane but also two-dimensional (2D) images in the coronal, sagittal, and even oblique or curved image planes. In addition, the usefulness of CBCT as a reliable tool for the presurgical measurement of the IAC region has been recently confirmed [11].

In Sudan, there's a lack of specific data on IAC morphometry. Given the country's ethnic diversity, local variations are expected. A morphometric study in the Sudanese population could provide essential data for safer dental and surgical interventions, improving patient outcomes. Hence, this study was conducted to determine the morphometric analysis of the IAC in a Sudanese population by using CBCT.

## Materials and methods

This descriptive-analytical cross-sectional study was conducted in the implantology center from 01.03.2022 to 31.03.2023. Ethical approval was obtained from the Research Ethics Committee of the National University, Sudan (approval number NU-REC/11-023/10). Informed consent was taken from the subjects. The research purpose and objectives were explained to participants in clear, simple, and understandable language for all the subjects. In total, 100 subjects were selected, and all patients in the implantology center underwent CBCT before planning treatment.

Inclusion criteria involved all patients of Sudanese ethnicity with healthy anatomical structure of the mandible whose age ranged between 18 and 65 years. Exclusion criteria involved non-Sudanese patients with mandibular trauma and patients aged less than 18 years or more than 65 years. Patients diagnosed with oral tumors or a history of maxillofacial surgeries were also not included in the study.

The examinations of the mandible and IAC were performed using CBCT at our implantology center. The Blue Sky Plan software (Blue Sky Bio LLC, Grayslake, US) was used to measure all the parameters. The Blue Sky Plan is a computer-based software for viewing and reformatting images created by computerized tomography and can be used for virtual implant treatment planning and surgical guide fabrication. Before loading the CBCT images, the software was calibrated for accurate bone measurement and nerve tracing. The software’s automatic nerve detection feature was utilized to trace the mandibular nerve from the mandibular foramen to the mental foramen. In order to confirm that the automatically traced nerve path was accurate, it was compared to the visible anatomical structures in the CBCT images. When necessary, the nerve path was manually adjusted to make sure it followed the right path.

The Ruler tool within the software was used to measure the bone dimensions at specific points of interest. The Ruler tool measures the bones and automatically identifies nerves from beginning to end. It works by tracing the nerve from the ramus area to the mental end. To measure bone in any location using the tool, an opening must be made in the sagittal cut to assess the bone in any area with the Ruler. The Ruler can record bone thickness, height, and other relevant measurements on CBCT images. For our study, all bone measurements were recorded, and the nerve path was traced. All measured data were accurately labeled and saved for analysis.

**Figure 1:**
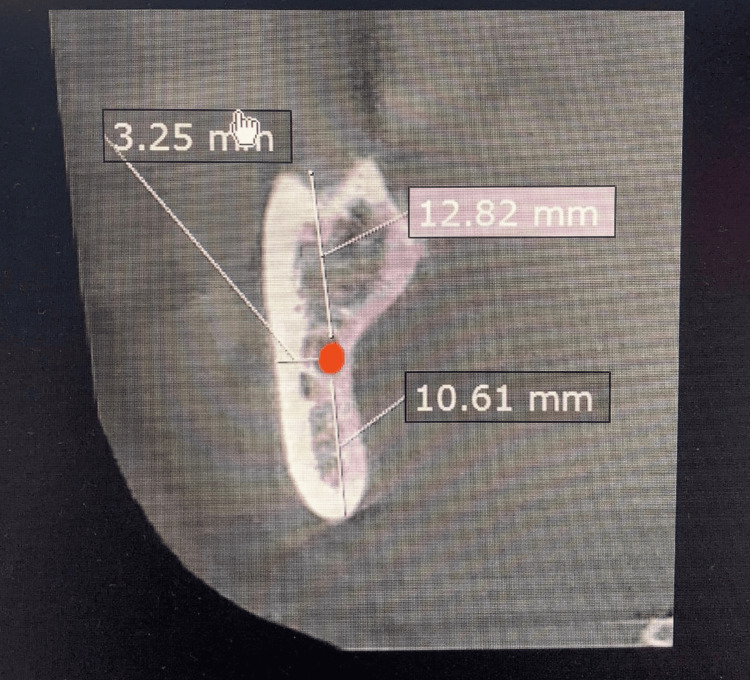
A sagittal cut from the original CBCT image showing the measurements of the mandibular ramus by Ruler tool surrounding the IAC. Ramus vertico occlusal (12.82 mm), ramus vertico apical (10.61 mm), and ramus horizontal buccal (3.25 mm). CBCT: cone-beam computed tomography; IAC: inferior alveolar canal

**Figure 2:**
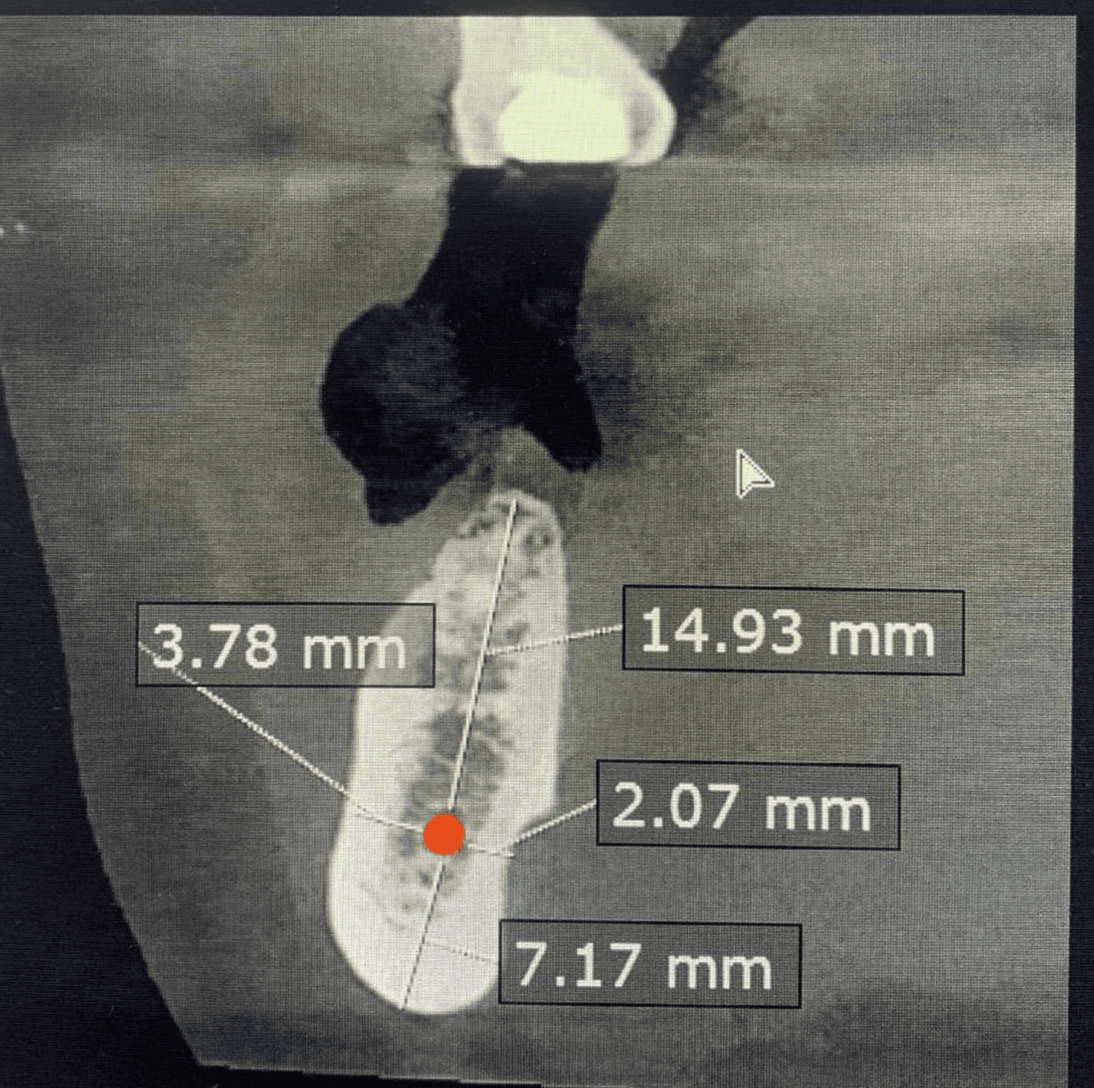
A sagittal cut from the original CBCT image showing the measurements of the body of the mandible by Ruler tool surrounding the IAC. Body vertico occlusal (14.93 mm), body vertico apical (7.17 mm), body horizontal buccal (3.78 mm), and body horizontal lingual (2.07 mm). CBCT: cone-beam computed tomography; IAC: inferior alveolar canal

We measured specific dimensions of the mandible surrounding the IAC on both the right and left sides across three distinct regions. The vertical dimension was assessed above and below the canal in the ramus, body, and mental areas. In the ramus region, measurements included the ramus vertico apical (VA) and ramus vertico occlusal (VO), as shown in Figure 1. For the body of the mandible, the body VA and body VO were recorded (Figure 2). In the mental area, the vertical dimensions were labeled as mental VA and mental VO.

**Figure 3:**
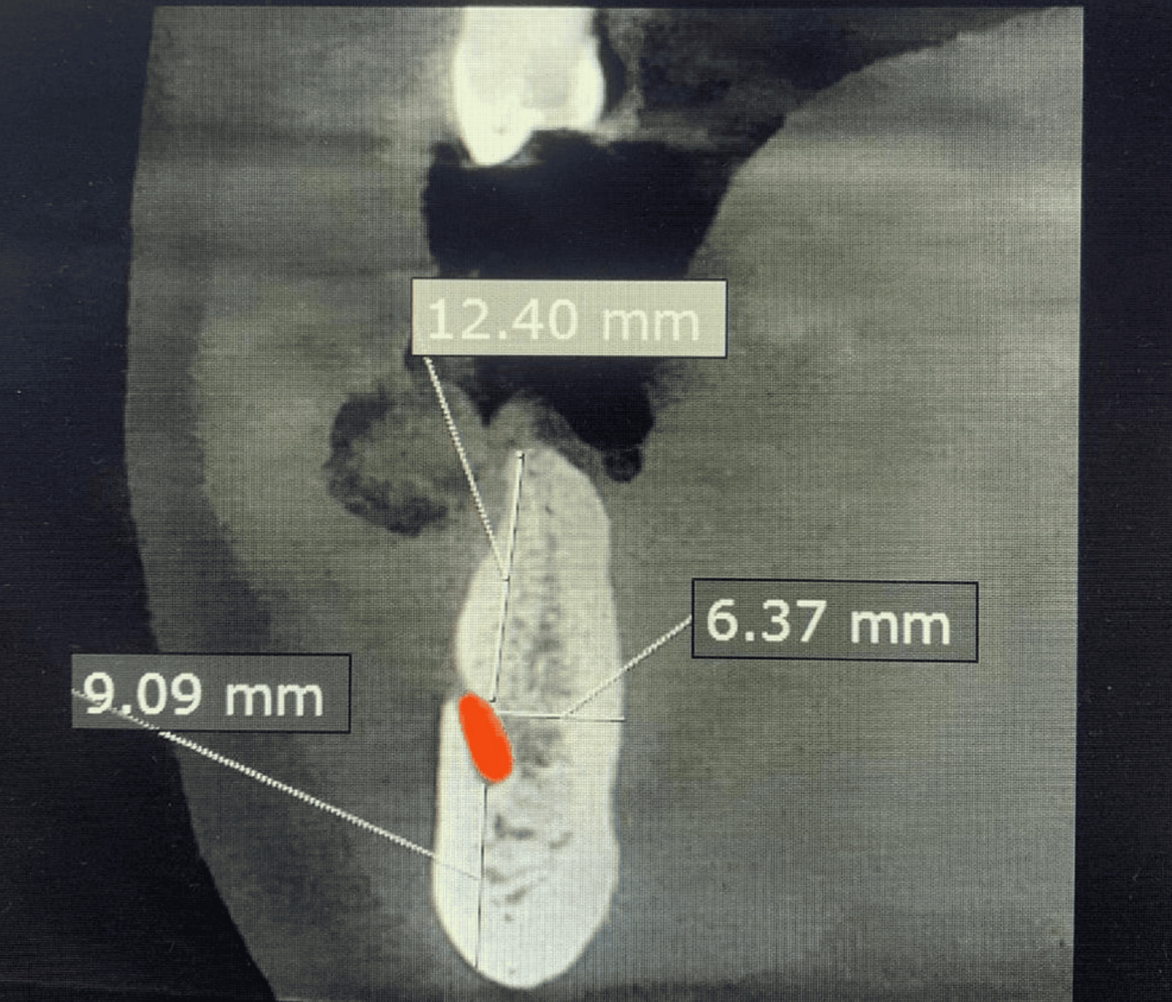
A sagittal cut from the original CBCT image showing the linear measurements of the various dimensions of the mandible surrounding the mental foramen. Mental vertico occlusal (12.4 mm), mental vertico apical (9.09 mm), and mental horizontal (6.37 mm). CBCT: cone-beam computed tomography; IAC: inferior alveolar canal

The horizontal dimension of the mandible surrounding the IAC was also measured in these same regions. In the ramus, the horizontal dimensions were identified as ramus horizontal lingual (HL) and ramus horizontal buccal (HB). For the body, measurements included body HB and body HL. Finally, in the mental area, the mental horizontal dimension was assessed (Figure 3).

The collected data were organized in a Microsoft Office Excel spreadsheet version 2010 (Microsoft Corp., Redmond, US). All statistical analyses were carried out using IBM SPSS Statistics for Windows version 20.0 (IBM Corp., Armonk, US). Descriptive statistics were used to describe the data in terms of frequency distribution, as well as mean and standard deviation. An independent t-test was used to assess if there were any significant differences noted between male and female patients.

## Results

Of the total participants, 84% were female and 16% were male. A significant difference was observed on the left side of the ramus VO according to gender (p-value =0.039*), whereas an insignificant difference was noted on the right side (p-value = 0.175). When comparing the ramus VA on the right side by gender, we discovered an insignificant difference (p-value = 0.829), and a similar insignificant difference on the left side of the ramus VA was also noted (p-value = 0.079). There was a significant difference (p-value = 0.003*) between the right and left sides of the ramus HB according to gender, whereas the left side showed an insignificant difference (p-value = 0.443). We also discovered that there was an insignificant difference in the ramus HL on both the right and left sides (p-values = 0.324 and 0.934, respectively). There was an insignificant difference on both sides of the body VO according to gender (p-value = 0.104 for the right side and p-value = 0.125 for the left side). When evaluating the right and left sides of the body VA according to gender, we discovered a minor difference (p-values = 0.390 and 0.620, respectively).

We noticed that there was an insignificant difference in the HB on the right side of the body (p-value = 0.309) and an insignificant difference in the HB on the left side of the body according to gender (p-value = 0.229). When comparing the body HL on the right side by gender, we discovered that there was an insignificant difference (p-value = 0.070), and a similar insignificant difference (p-value = 0.554) was noted on the left side of the body HL.

When we compared mental VO on the right and left sides according to gender, we observed no significant difference (p-values = 0.693 and 0.903, respectively). When comparing the right and left sides of the mental VA according to gender, we observed no significant difference as well in this morphometric parameter (p-values = 0.270 and 0.311, respectively). When we compared the mental horizontal on the right side according to gender, we discovered an insignificant difference (p-value = 0.718); similarly, an insignificant difference was noted on the left side of the mental horizontal (p-value = 0.553). The results are depicted in detail in Tables 1-3.

**Table 1:** Comparison of ramus VO, VA, HB, and HL on the right and left sides according to gender. (*) p values of 0.039 and 0.003 (<0.05), denoting a significant difference on the right side of the ramus HB in male and female patients. VO: vertico occlusal; VA: vertico apical; HB: horizontal buccal; HL: horizontal lingual

Site of the measure	Right		p-value	Left		p-value
	Male	Female		Male	Female	
Ramus VO	20.26 ± 4.7	18.79 ± 3.8	0.175	21.82 ± 4.0	19.155 ± 4.7	0.039 (*)
Ramus VA	11.06 ± 3.5	11.3 ± 4.4	0.829	14.3 ± 14.3	10.7 ± 4.1	0.079
Ramus HB	5.27 ± 1.30	4.1 ± 1.32	0.003 (*)	4.7 ± 1.48	4.4 ± 1.78	0.443
Ramus HL	2.36 ± 1.31	2.67 ± 1.14	0.324	2.60 ± 1.40	2.63 ± 1.20	0.934

**Table 2:** Comparison of body VO, VA, HB, and HL on the right and left sides according to gender. VO: vertico occlusal; VA: vertico apical; HB: horizontal buccal; HL: horizontal lingual

Site of the measure	Right		p-value	Left		p-value
	Male	Female		Male	Female	
Body VO	18.7 ± 3.59	17.2 ± 3.21	0.104	18.8 ± 3.88	17.2 ± 3.7	0.125
Body VA	8.5 ± 1.64	8.0 ± 1.97	0.390	7.3 ± 1.55	7.14 ± 1.96	0.620
Body HB	4.73 ± 1.78	5.13 ± 1.37	0.309	4.7 ± 1.69	5.13 ± 1.12	0.229
Body HL	4.82 ± 1.70	3.34 ± 1.11	0.070	3.66 ± 1.57	3.45 ± 1.25	0.554

**Table 3:** Comparison of mental VO, VA, and horizontal on the right and left sides according to gender. VO: vertico occlusal; VA: vertico apical

Site of the measure	Right		p-value	Left		p-value
	Male	Female		Male	Female	
Mental VO	16.9 ± 5.0	17.33 ± 3.6	0.693	17.2 ± 3.48	17.3 ± 2.95	0.903
Mental VA	10.7 ± 1.88	10.12 ± 2.1	0.270	10.44 ± 1.7	9.82 ± 2.2	0.311
Mental horizontal	5.64 ± 1.42	5.46 ± 1.92	0.718	5.68 ± 1.66	5.40 ± 1.77	0.553

## Discussion

The IAC is a canal within the mandible that begins in the mandibular foramen. It runs obliquely downward and forward in the ramus and then horizontally forward in the body. It carries an inferior alveolar neurovascular bundle [12]. Different anatomical variations may occur in IAC, such as bifid or trifid, resulting from embryological development errors. Knowledge of the anatomy of the IAC and its location is of great importance to the oral and maxillofacial surgeon. Several studies have been conducted using various radiological techniques, such as traditional CT scans and morphological analyses in cadavers, to investigate the architecture of the IAC [3,5,13].

The current study explored various mandibular measurements to assess its morphology and look for any gender differences in the Sudanese population by using the CBCT. CBCT has been reported as a well-suited imaging modality for the craniofacial area. It provides clear and accurate images of structures and, therefore, is extremely useful for assessing the bone component. As the resultant images displayed are often corrected for magnification, accurate measurements can be derived from the reformatted three-dimensional (3D) data [13]. While comparing the findings in this study with previous studies, for the ramus VO measurement, our study found an insignificant difference on the right side (p=0.175) but a significant difference on the left side (p=0.039*). This aligns partially with the findings of Direk et al. [14] who reported side-dependent gender differences, while Ayad et al. [15] focused on other mandibular dimensions without specifically addressing VO measurement.

In terms of the ramus VA measurement, both sides showed insignificant differences (p=0.1829 on the right side and p=0.079 on the left side). This contrasts with Assari et al. [16] who observed more pronounced gender differences in older populations, suggesting that age and population-specific factors might influence these measurements.

The ramus HB measurement revealed a significant difference on the right side (p=0.003*) but an insignificant difference on the left side (p=0.443). This finding is consistent with Direk et al. [14] who found significant gender differences in horizontal dimensions, particularly on the right side, indicating possible functional or habitual factors affecting this measurement. Additionally, there were insignificant differences between the left and right sides of the ramus HL measurement (p=0.934 and p=0.324, respectively). While Assari et al. [16] and Hettiarachchi et al. [17] made significant suggestions about gender-specific morphological or functional differences in the lingual side of the mandible, their findings and our data for the ramus HL were not comparable indicating a different anatomy of mandible in the Sudanese population.

The body VO measurement showed insignificant differences on both sides (p=0.104 on the right side and p=0.125 on the left side), contrasting with some previous studies that observed gender differences in other mandibular regions. This suggests that the VO dimension may not be a primary marker of sexual dimorphism in the studied population. Similarly, the body VA measurement presented insignificant differences on both sides (p=0.390 on the right side and p=0.620 on the left side), indicating that this dimension might not be highly sensitive to gender-related variations, possibly due to the younger age range or specific population characteristics. The body HB measurement also showed insignificant differences on both sides (p=0.309 on the right side and p=0.229 on the left side), which could indicate a more uniform distribution of this dimension across genders, as suggested by Ayad et al. [15].

For the body HL measurement, the study found an insignificant difference on the right and left sides (p=0.070 and p=0.554, respectively). These observations in our cohort were not in concordance with the findings of Assari et al. [16] and Hettiarachchi et al. [17] who noted significant gender-specific variations in the lingual aspect of the mandible. Finally, the mental VO measurement showed insignificant differences on both sides (p=0.693 on the right and p=0.903 on the left), suggesting that this dimension may not be a key indicator of sexual dimorphism in the studied population, consistent with the variability reported by Direk et al. [14].

The human mandible is one of the extensively studied bones for sexual dimorphism and age-related remodeling. Mandibular size and shape differ significantly between male and female populations [8]. Different mandibular measurements like bigonial breadth, bicondylar breadth, and mandibular length have been studied by many authors as the most discriminatory parameters between male and female participants [18,19], while some studies have also reported a significant sex-related difference in the mandibular canal position [20].

Overall, these findings highlight the complexity of gender differences in mandibular measurements, with some dimensions showing significant variability depending on side, population, and possibly age, while others remain consistent across genders. Our study contributes to the understanding of mandibular morphometrics in the Sudanese population, providing insights that may differ from those observed in other ethnic groups

Study limitations

This study has a few limitations. First, the sample size was relatively small, and it was restricted to one implantology center, which might limit the extension of results to the general population and provide a rough morphometric estimate of IAC in the Sudanese population. Second, there was a huge gender difference; this could be attributed to the high awareness of women's dental hygiene, cosmetic reasons, and increased sensitivity to health-related issues in the female population.

## Conclusions

This study investigated the morphometric analysis in various mandibular measurements in a Sudanese population, revealing both significant and insignificant differences depending on the specific dimension and side of the mandible. Most of the morphometric parameters were similar on both sides of the mandible and showed no significant differences in the male and female populations. Nonetheless, there were notable gender variations on the right and left sides of the ramus VO and ramus HB measurements, indicating that these metrics could be useful in determining gender in this population. Nevertheless, this significant gender difference could also help in determining the extent of needle insertion when giving inferior alveolar blocks. However, other crucial measurements such as ramus VA and body HB showed insignificant differences, indicating that these dimensions may not be reliable markers of sexual dimorphism in this demographic.

In clinical practice, our findings are important for preventing neurosensory sequelae in mandibular surgeries, as maxillofacial surgeons and dentists need to administer anesthesia through the inferior alveolar block. For this, they must know the anatomical location of the IAC and the mental foramen. Our findings offer valuable insights into the morphometric parameters of the IAC. However, the observed variations highlight the need for further investigation into the underlying factors that influence these differences, including age, ethnicity, and functional adaptation.
